# Salinity-driven canthaxanthin enhancement in *Chlorosarcinopsis* PY02: a practical spot test for microalgal bioprocess optimization

**DOI:** 10.5114/bta/214478

**Published:** 2025-12-23

**Authors:** Thanyanan Wannathong Brocklehurst, Nipawan Puedsing, Theera Thurakit, Phinyarat Sensupa, Saranyaporn Maksap, Orawan Borirak

**Affiliations:** 1 Department of Biology, Faculty of Science, Silpakorn University, Nakhon Pathom, Thailand; 2 Department of Applied Microbiology, Institute of Food Research and Product Development, Kasetsart University, Bangkok, Thailand; 3 Algal and Cyanobacterial Research Laboratory, Faculty of Science, Chiang Mai University, Chiang Mai, Thailand; 4 Department of Microbiology, Faculty of Science, Silpakorn University, Nakhon Pathom, Thailand

**Keywords:** sustainable algal biotechnology, natural antioxidants, abiotic stress response, salinity-driven pigment biosynthesis, ketocarotenoids

## Abstract

**Background:**

As society rapidly ages, the escalating global demand for natural, high-value antioxidants – particularly ketocarotenoids such as canthaxanthin – is driving intensive research into their sustainable bioproduction. This study investigates the potential of the green microalga *Chlorosarcinopsis* PY02 as a novel microbial cell factory for enhanced ketocarotenoid production under abiotic stress conditions.

**Materials and methods:**

We optimized bioprocess parameters using a simple, spot-test-based high-throughput screening technique, evaluating algal growth and pigment accumulation on tris acetate phosphate agar supplemented with various sodium chloride concentrations (0–15 g/l).

**Results:**

Peak canthaxanthin content (294.55 µg/g dry weight) was observed at 10 g/l NaCl, while biomass yield was highest at 12 g/l. Combining salt stress with a 50% nitrogen reduction increased total carotenoid productivity (highest with 10 g/l NaCl: 3.10 mg/l) but did not enhance canthaxanthin levels; the salt-only treatment produced the highest canthaxanthin yield (0.80 mg/l). Pigment identification and quantitative profiling were performed using thin-layer chromatography and spectrophotometry, confirming the efficiency of the production process.

**Conclusions:**

These findings highlight *Chlorosarcinopsis* PY02 as a promising candidate for sustainable, large-scale production of ketocarotenoids. The study also demonstrates a cost-effective and scalable approach for inducing carotenoid biosynthesis in *Chlorosarcinopsis* PY02, with strong relevance for sustainable pigment production. The simple visual screening method provides a practical tool for preliminary strain and condition optimization in microalgal bioprocess development.

## Introduction

Due to current fast-paced lifestyles, work-related stress, air pollution, dietary habits, and UV radiation exposure, various diseases have become increasingly prevalent, primarily as a result of free radical generation (Mustafa 2024). In addition to physical exercise, the consumption of antioxidant-rich dietary supplements has gained popularity as a preventive measure. This is supported by market forecasts, which indicate sustained interest in antioxidant-rich food supplements, particularly “ready-to-eat carotenoid-rich products” (Yaqoob et al. 2021). This trend is rapidly increasing (Ambati et al. 2019; Bogacz-Radomska et al. 2020).

Carotenoids – especially ketocarotenoids – represent a well-studied group of antioxidants with consistently demonstrated health benefits. Ketocarotenoids are a subset of carotenoids characterized by a ketone functional group (C = O) within their molecular structure; astaxanthin and canthaxanthin are well-recognized examples. These compounds are known for their strong antioxidant properties (Kumar et al. 2022), making astaxanthin and canthaxanthin particularly sought after for their potential applications in human health and as high-value biochemicals (Patil et al. 2022; Gaur and Bera 2023). Extensive research has investigated the roles of ketocarotenoids in preventing various diseases and health conditions in humans, including cancer (Hamidi et al. 2020), cardiovascular diseases (Fassett and Coombes 2012), inflammation, and immune stimulation (Park et al. 2010). These conditions are of significant concern in an aging society, prompting continued efforts to identify effective preventive strategies.

In light of these benefits, producers are actively seeking natural carotenoid sources suitable for industrial-scale production. Among these, small green microalgae such as *Haematococcus pluvialis* and *Dunaliella salina* have garnered substantial attention. Research aimed at improving and developing methods for carotenoid production from these microalgae has been ongoing (Oslan et al. 2021; da Silva et al. 2021). Much of this work focuses on determining optimal conditions for carotenoid production, including the effects of nitrogen reduction (Coulombier et al. 2020; Jo et al. 2020), increased salinity (Elloumi et al. 2020), light intensity (Faraloni et al. 2021), and other related parameters (Minhas et al. 2016). These investigations have confirmed that ketocarotenoids from *H. pluvialis* exhibit higher antioxidant activity than their synthetic analogs (Khalid et al. 2019). Understanding ketocarotenoid biosynthesis pathways is essential for developing more efficient microbial cell factories through metabolic engineering and genetic manipulation (Chen et al. 2022; Cui et al. 2020; Kopec and Failla 2018).

While *H. pluvialis* is a well-established producer, its slow growth rate, large cell size, and complex cultivation requirements limit its industrial scalability (Cui et al. 2020; Oslan et al. 2021). This has driven extensive efforts to identify alternative microalgal strains with improved growth kinetics and production efficiency (Aburai et al. 2015; Seger et al. 2023).

Based on these considerations, this study aims to investigate the effects of chloride salt (NaCl) on the growth and carotenoid accumulation of the small green microalga *Chlorosarcinopsis* PY02, a microalga known for efficiently synthesizing and accumulating ketocarotenoids under laboratory cultivation at the Department of Biology, Faculty of Science, Silpakorn University.

## Materials and methods

### Cultivation of green microalga Chlorosarcinopsis PY02

The cultivation and maintenance of the *Chlorosarcinopsis* PY02 microalga were conducted under laboratory conditions (Cherdchukeattisak et al. 2018). The microalga was cultured on tris acetate phosphate (TAP) medium supplemented with 2% agar. The algal plates were incubated at 25 ± 1°C under continuous white light, with a light intensity of 60 µmol m^-2^ s^-1^. These conditions were established to provide a starting culture for subsequent experiments.

### Effects of NaCl on growth and carotenoid accumulation in Chlorosarcinopsis PY02

The microalga’s ability to grow on solid TAP medium supplemented with varying NaCl concentrations – 0, 3, 5, 10, 12, 15, 20, and 35 g/l (with standard TAP medium serving as the control) – was assessed using a spot-test technique. Briefly, microalgal cells initially cultivated on solid agar were scraped, collected, and weighed as fresh weight (FW). The cells were then suspended in liquid TAP medium at a density of 20 mg FW per milliliter (mg FW/ml). Subsequently, 50 µl of the microalgal suspension was spotted onto solid TAP medium containing different NaCl concentrations, and the microalgae were cultured as described above. This experiment was performed in quintuplicate.

The effects of salt on growth were documented through photographs taken at five-day intervals over a total of 70 days to capture the stable NaCl-induced green-to-orange/red phenotype. After this period, the microalgal cells were harvested and freeze-dried for subsequent carotenoid extraction and analysis.

### Effects of NaCl supplementation combined with nitrogen reduction on growth and carotenoid accumulation in Chlorosarcinopsis PY02

The NaCl concentrations identified from the previous experiment on salt effects were further examined in combination with a 50% reduction in nitrogen content in liquid TAP medium (total volume: 100 ml). Nitrogen reduction was achieved by decreasing the concentration of the sole nitrogen source (NH_4_Cl) from 7.5 to 3.75 mM, while all other TAP constituents remained unchanged. This experiment was carried out in five replicates. Observations were recorded at 7-day intervals over a period of 28 days. At the end of the experiment, the microalgal cells were harvested and freeze-dried for carotenoid analysis.

### Extraction and separation of carotenoids from Chlorosarcinopsis PY02

Dried algal cells (8 mg) were finely ground with sea sand (Nr. 41 845, Ferak Berlin GmbH) and then weighed in a 2 ml microcentrifuge tube to achieve a total mass ten times the algal weight. The sample was homogenized into a uniform paste using 400 µl of distilled water and 400 µl of methanol. The mixture was vigorously shaken and incubated in the dark for 1 h. Following this, 800 µl of chloroform was added, and the solution was shaken vigorously before centrifugation at 13,000 revolutions per minute (rpm) for 5 min. The nonaqueous phase was collected and dried at room temperature in the dark until a constant weight was reached (Cherdchukeattisak et al. 2018).

Carotenoid separation from the algal extracts was performed according to previously described methods (Grung et al. 1992). The mobile phase (acetone : hexane, 25 ml : 75 ml) was prepared, and the algal extract was dissolved in 15 µl of chloroform and gently mixed. Subsequently, 25–30 µl of petroleum ether was added, and the solution was mixed thoroughly before being spotted onto thin-layer chromatography (TLC) plates. The Rf (retention factor) values for the carotenoids were calculated by comparison with β-carotene (C4582-5MG, Sigma-Aldrich) and canthaxanthin (32993-5MG, Fluka) standards.

### Quantification of total carotenoids and canthaxanthin in Chlorosarcinopsis PY02

Total carotenoid content was determined by measuring light absorption at wavelengths of 470, 644, and 662 nm in the extracted algal solution using a spectrophotometer. To prepare the sample, 1 ml of petroleum ether was added to the *Chlorosarcinopsis* PY02 extract, mixed thoroughly, and centrifuged at 6,500 rpm for 2 min. The clear supernatant was then transferred into a new tube, and the volume was adjusted to 2 ml with additional petroleum ether. Petroleum ether served as the blank. Absorbance at the specified wavelengths was recorded, and total carotenoid content and productivity were calculated using the following equation (1) described by Dere et al. (1998):


$$\begin{array}{c} \text{Total carotenoid }(\mu \text{g}/\text{ml})=\\ 1000{\text{A}}_{470}-1.280\;(10.05{\text{A}}_{662}-0.766{\text{A}}_{644})-\\ -56.7\;(16.37{\text{A}}_{644}-3.140{\text{A}}_{662})/230 \end{array}$$ (1)

where *A_470_, A_644_*, and *A_662_* represent the absorbance at 470 nm, 644 nm, and 662 nm, respectively.

Solvent note: The Dere et al. (1998) multi-wavelength equation was originally derived for diethyl ether; a chlorophyll-corrected, multi-wavelength alternative for petroleum ether is not available. Therefore, we applied the Dere formulation to petroleum-ether extracts to preserve internal comparability, interpreting totals as relative differences among treatments rather than solvent-absolute values. Where relevant, totals are also reported as β-carotene equivalents based on petroleum-ether extinction coefficients.

For canthaxanthin determination, the TLC band corresponding to the standard position of canthaxanthin in the *Chlorosarcinopsis* PY02 extract was carefully scraped and transferred into a 1 ml microcentrifuge tube. A volume of 300 µl of chloroform was added to extract the pigment, followed by thorough mixing. Then, 1000 µl of petroleum ether was added, and the mixture was shaken again. The solution was centrifuged at 6,500 rpm for 2 min. The clear supernatant was transferred into a glass cuvette, and the volume was adjusted to 2 ml with petroleum ether. Canthaxanthin content was determined spectrophotometrically from the petroleum-ether extracts.

Initial estimates were guided by the Beer-Lambert law (Calloway 1997), using the extinction coefficient for canthaxanthin in petroleum ether described by Davies (1965). To ensure accuracy and account for laboratory-specific conditions, a standard curve was generated using known concentrations of pure canthaxanthin standard (Fluka, Cat. No. 32993-5MG) dissolved in petroleum ether. Absorbance at 466 nm was recorded. A linear regression analysis yielded the following empirical equation (2) for canthaxanthin quantification:


$$\text{Canthaxanthin }(\mu \text{g}/\text{ml})=({\text{A}}_{466}+0.0002)/0.22$$ (2)

where *A_466_* represents the absorbance at 466 nm. This standard curve demonstrated excellent linearity with a coefficient of determination (*R*^2^) of 0.99.

### Statistical data analysis

In experiments where quantitative data were collected with at least three replicates, results were expressed as mean ± SD. Differences among treatments were assessed using one-way ANOVA (IBM SPSS Statistics v23), followed by Duncan’s multiple range test. Means denoted by different letters were considered significantly different (*p* < 0.05).

## Results and discussion

### Salt tolerance and enhanced carotenoid production in Chlorosarcinopsis PY02

**Figure 1:**
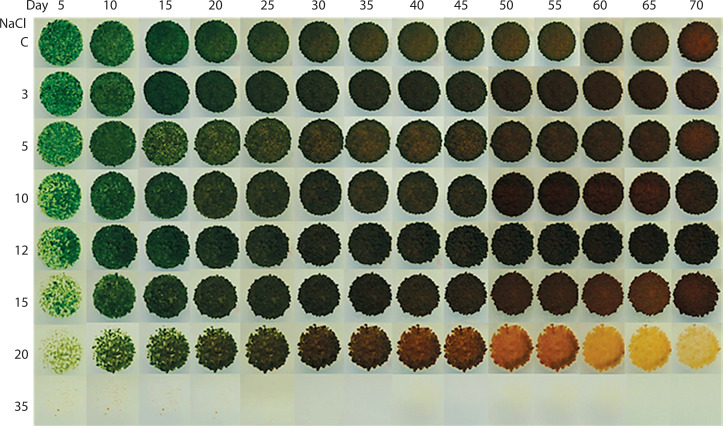
Impact of salt on the growth of *Chlorosarcinopsis* PY02 on solid tris acetate phosphate medium supplemented with sodium chloride using the spot test technique. C – control, and numbers 3, 5, 10, 12, 15, 20, and 35 – NaCl concentration (in g/l)

*Chlorosarcinopsis* PY02 demonstrated a remarkable ability to tolerate a wide range of salinity levels, high-lighting its adaptability. Algal growth and carotenoid production increased substantially between 0 and 15 g/l of salt concentration, with the most pronounced growth observed within this range. At 20 g/l, algal growth was notably reduced, and at 35 g/l, growth ceased entirely. A visible shift in chlorophyll colour – from green to dark brown-orange – occurred around days 20–25 of cultivation, and by day 70, the algal cells exhibited a distinct orange coloration (Figure 1). These discoveries validated that this *Chlorosarcinopsis* PY02 soil alga possesses the ability to withstand a specific spectrum of salt concentrations, and the results also suggested a correlation between the algal response to salt stress and the increased production of carotenoids. This observation underscores the simplicity, efficiency, and resource-saving advantages of the spot-test technique, particularly as a practical initial screening tool for identifying suitable algal strains or optimal conditions for carotenoid production across different applications and scales.

**Figure 2:**
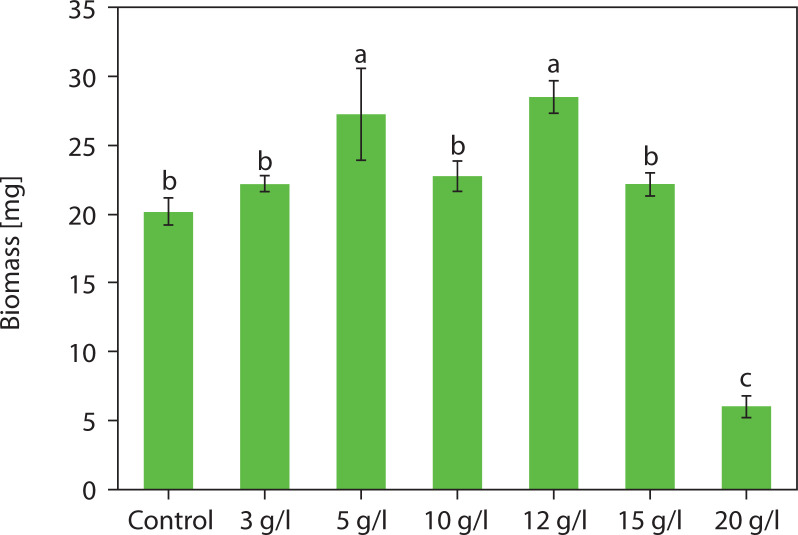
Impact of salt on the biomass of *Chlorosarcinopsis* PY02 on solid tris acetate phosphate (TAP) medium supplemented with sodium chloride. Control – normal TAP, 3 g/l – TAP + 3 g/l NaCl, 5 g/l – TAP + 5 g/l NaCl, 10 g/l – TAP + 10 g/l NaCl, 12 g/l – TAP + 12 g/l NaCl, 15 g/l – TAP + 15 g/l NaCl, 20 g/l – TAP + 20 g/l NaCl, and different lowercase letters indicate significant differences between treatments (*p* < 0.05)

Dry biomass analysis revealed that salinity had a significant effect on biomass production in *Chlorosarcinopsis* PY02 at various levels (Figure 2). Notably, at salt concentrations of 5 and 12 g/l, biomass production increased significantly (*p* < 0.05), reaching 1.3- and 1.4-fold higher values than the control. The average dry weight (DW) peaked at 27.25 ± 6.64 mg and 28.48 ± 2.34 mg, respectively. In contrast, a salinity level of 20 g/l led to an approximately 3.3-fold reduction in average biomass compared to the control, indicating that this higher concentration may induce stress severe enough to hinder the growth of *Chlorosarcinopsis* PY02.

### Influence of NaCl supplementation on accumulated carotenoid types in Chlorosarcinopsis PY02

**Figure 3:**
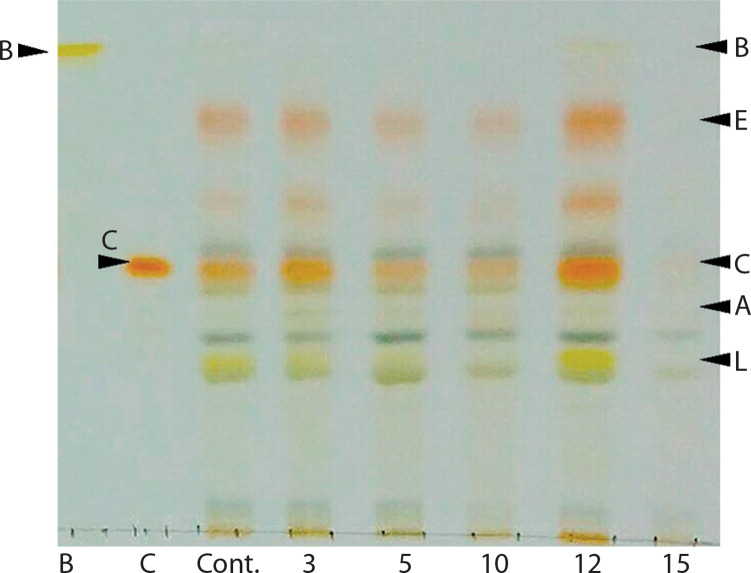
Thin-layer chromatogram (TLC) of *Chlorosarcinopsis* PY02 crude extract grown on tris acetate phosphate agar medium supplemented with various concentrations of chloride salt. B at the TLC spot sample – β-carotene standard, C at the TLC spot sample – canthaxanthin standard, Cont. – control, numbers 3, 5, 10, 12, and 15 – NaCl concentration in g/l. Bands labeled A (astaxanthin), E (echinenone), and L (lutein) are assigned based on the previously validated carotenoid profile of this strain by ultra-performance liquid chromatography (Cherdchukeattisak et al. 2018). Only β-carotene and canthaxanthin were confirmed with authentic TLC standards in this study

The color changes observed in the spots – transitioning from light green to green and eventually to orange – were indicative of alterations in carotenoid composition during algal growth. This pattern was further confirmed using TLC, following the method developed by Grung et al. (1992). The carotenoids identified included β-carotene, lutein, echinenone, canthaxanthin, and astaxanthin, as illustrated in Figure 3. These findings are consistent with a previous study by Cherdchukeattisak et al. (2018), which used ultra-performance liquid chromatography. Specifically, the green phase in *Chlorosarcinopsis* PY02 cells corresponded to lutein as the dominant carotenoid, while the transition to orange reflected the accumulation of ketocarotenoids, particularly echinenone, 3-OH-echinenone, and canthaxanthin.

Regarding total carotenoid content, the introduction of salt into the culture system had a substantial effect. Total carotenoid content increased by approximately 1.2 to 1.4 times compared with the control (Table 1). This enhancement was especially pronounced in cultures grown in TAP medium supplemented with 5, 10, and 12 g/l NaCl, which produced significantly higher total carotenoid levels (*p* < 0.05), with the highest at 27.50 ± 6.01 µg with NaCl at 12 g/l. This increase was statistically significant (*p* < 0.05), with carotenoid contents of 914.03 ± 86.63, 1,010.45 ± 67.45, and 934.78 ± 135.57 µg/g DW at 5, 10, and 12 g/l NaCl, respectively.

**Table 1:** Carotenoid content and production in *Chlorosarcinopsis* PY02 grown on tris acetate phosphate (TAP) agar medium supplemented with chloride salt

Algal media	Carotenoid content [µg/g dry weight]		Carotenoid production [µg]	
	Total carotenoid	Canthaxanthin	Total carotenoid	Canthaxanthin
TAP (control)	716.92 ± 43.25^b^	105.91 ±12.81^c,d^	15.09 ± 1.90^b^	2.23 ± 0.32^c^
TAP + NaCl 3 g/l	650.22 ± 23.27^b^	112.73 ± 26.57^c,d^	14.12 ± 0.46^b^	2.46 ± 0.64^c^
TAP + NaCl 5 g/l	914.03 ± 86.63^a^	154.39 ± 27.77^c^	26.69 ± 6.85^a^	4.60 ±1.67^b^
TAP + NaCl 10 g/l	1,010.45 ± 67.45^a^	294.55 ± 66.35^a^	23.80 ± 3.16^a^	6.86 ±1.30^a^
TAP + NaCl 12 g/l	934.78 ± 135.57^a^	232.05 ± 27.77^b^	27.50 ± 6.01^a^	6.74 ± 0.44^a^
TAP + NaCl 15 g/l	439.51 ± 80.70^c^	60.45 ± 16.74^d^	10.06 ± 2.29^b^	1.39 ± 0.45^c^

Different lowercase letters within a column indicate significant differences (p < 0.05).

In contrast, salt concentrations of 3 and 15 g/l led to decreases in total carotenoid content – approximately 0.6 to 0.9 times lower than the control (716.92 ± 43.25 µg/g DW).

Analysis of canthaxanthin content in the *Chlorosarcinopsis* PY02 samples revealed clear trends in response to salinity. Most salt-supplemented treatments showed substantial increases in canthaxanthin content. The most pronounced increase – up to 2.7-fold – occurred at 10 g/l NaCl, and this rise was statistically significant (*p* < 0.05), with a canthaxanthin content of 294.55 ± 66.35 µg/g DW. At 3 g/l, canthaxanthin levels did not differ significantly from the control. Conversely, at 15 g/l, canthaxanthin content decreased by 1.75-fold compared with the control, reaching 105.91 ± 12.81 µg/g DW.

In the field of algae cultivation aimed at obtaining secondary metabolites, understanding the impact of stress levels is essential (Shi et al. 2020; Oslan et al. 2021). Different algal species exhibit diverse responses to salinity, with *Chlorosarcinopsis* PY02 thriving within a range of 0–15 g/l NaCl, which supports optimal biomass production – particularly at 5–12 g/l. These findings emphasize the importance of tailoring salinity levels to the specific requirements of each algal species to maximize yield and productivity.

For example, the halophile *Dunaliella salina* tolerates extremely high salinity levels, approaching saturation (5.5 M or 321.4 g/l NaCl) (Yao et al. 2025). In contrast, *Chlorella protothecoides* shows moderate halotolerance, maintaining growth and increasing lipid formation at 30 g/l NaCl (Wang et al. 2016). In *H. pluvialis*, low NaCl concentrations (12.5 mg/l) have been reported to enhance biomass formation, while adding 2 g/l NaCl in a two-stage cultivation process nearly doubled astaxanthin accumulation (Li et al. 2022).

Salt stress affects microalgae at multiple levels, including physiological traits, gene expression, and metabolic pathways. It can reduce cell growth, decrease chlorophyll content, inhibit photosynthesis, and induce morphological changes. However, salt stress is widely recognized as an effective strategy for enhancing carotenoid accumulation due to its low cost, simplicity, and high profitability (Ren et al. 2021). In general, moderate salt stress can increase biomass productivity and stimulate carotenoid accumulation, depending on microalgae species, while strict stress will be toxic and even fatal to microalgae. As both high cell density and carotenoid productivity are desirable for biotechnological goals, an optimal salt concentration should be chosen cautiously according to different cultivation goals and microalgae species. Two-stage or multi-stage cultivation and strategies combining salinity with other stress factors also have potential in microalgae cultivation for carotenoids (Fu et al. 2023).

### The impact of NaCl combined with nitrogen reduction on algal biomass production

**Figure 4:**
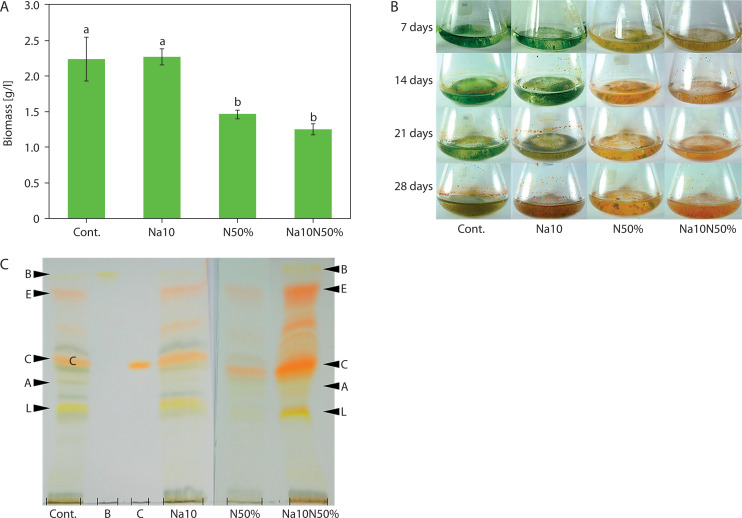
Effect of salt in combination with nitrogen reduction on biomass (**A**), culture colour (**B**), and carotenoids (**C**) of *Chlorosarcinopsis* PY02 in tris acetate phosphate liquid medium supplemented with chloride salt. Cont. – control, Na10 – NaCl 10 g/l, N50% – 50% nitrogen, Na10N50% – NaCl 10 g/l and 50% nitrogen, B at the thin-layer chromatography (TLC) spot sample – β-carotene standard, C at the TLC spot sample – canthaxanthin standard. Bands labeled A (astaxanthin), E (echinenone), and L (lutein) are assigned based on the strain ultra-performance liquid chromatography (Cherdchukeattisak et al. 2018). Different lowercase letters indicate significant differences between treatments (*p* < 0.05)

Comparative analysis of *Chlorosarcinopsis* PY02 biomass, measured as average DW (milligrams per liter; mg/l), revealed that TAP liquid medium with nitrogen reduction, as well as TAP medium supplemented with salt combined with nitrogen reduction, produced the greatest decreases in biomass – up to 1.8-fold lower than the control, which had an average DW of 2,234.0 ± 305.8 mg/l. However, the addition of salt alone to TAP medium did not significantly affect average DW compared to the control, as shown statistically in Figure 4A.

Color changes were observed across different media. In TAP medium with nitrogen reduction and TAP medium supplemented with both salt and nitrogen reduction (Figure 4B), algal cells shifted from green to reddish-orange by day 7, a faster transition than in the control. Although the combination of salt and nitrogen reduction did not alter the types of accumulated carotenoids (Figure 4C), it intensified the red carotenoid bands, indicating variations in pigment quantities. Canthaxanthin appeared as the most prominent band, becoming darker and thicker. These findings suggest that carotenoid production in *Chlorosarcinopsis* PY02 may be modulated by the combined influence of salinity and nitrogen availability, highlighting the need for further investigation into their interactive effects.

*Chlorosarcinopsis* PY02 cultures supplemented with salt and with salt combined with nitrogen reduction showed 2.0- and 2.2-fold increases, respectively, in total carotenoid content compared with the control group. The highest total carotenoid levels were observed in cultures grown in TAP medium supplemented with salt and in TAP medium supplemented with salt combined with nitrogen reduction, both showing statistical significance (*p* < 0.05), with values of 1,370.33 ± 253.12 and 1,437.98 ± 110.38 µg/g DW, respectively (Table 2). In contrast, TAP medium with 50% nitrogen reduction produced the lowest total carotenoid content at 526.01 ± 140.98 µg/g DW, with no significant difference compared to the control.

**Table 2:** Carotenoid content and production in *Chlorosarcinopsis* PY02 grown on tris acetate phosphate (TAP) liquid medium supplemented with chloride salt and salt combined with nitrogen reduction

Algal media	Carotenoid content [µg/g dry weight]		Carotenoid production [µg]	
	Total carotenoid	Canthaxanthin	Total carotenoid	Canthaxanthin
TAP	663.42 ± 123.67^b^	31.82 ±7.43^b^	1,505.83 ± 497.31^b^	70.83 ± 17.34^c^
TAP + Na10	1,370.33 ± 253.12^a^	352.95 ± 55.20^a^	3,100.64 ± 553.09^a^	800.54 ±133.93^a^
TAP + N50%	526.01 ± 140.98^b^	344.55 ± 55.84^a^	770.77 ± 219.41^c^	504.29 ± 92.26^b^
TAP + Na10N50%	1,437.98 ± 110.38^a^	55.23 ± 11.96^c^	1,798.56 ± 162.69^b^	69.50 ±17.96^c^

Na10 – NaCl 10 g/l, N50% – 50% nitrogen, Na10N50% – NaCl 10 g/l and 50% nitrogen.

Different lowercase letters within a column indicate significant differences (p < 0.05).

For canthaxanthin, the salt-supplemented and salt-plus-nitrogen-reduction treatments showed the most sub-stantial increases – 11.1- and 10.8-fold, respectively – relative to the control group.

However, supplementing salt in combination with nitrogen reduction did not enhance the canthaxanthin production potential of *Chlorosarcinopsis* PY02. Although the combined stress resulted in a statistically significant increase in total carotenoid content, the salt-only treatment was superior for overall canthaxanthin production (Table 2). Cultures grown in TAP medium supplemented with salt exhibited the highest total carotenoid production at 3,100.64 ± 553.09 µg, a 2.1-fold increase compared with the control (1,505.83 ± 497.31 µg). In contrast, TAP medium supplemented with salt combined with nitrogen reduction yielded 1,798.56 ± 162.69 µg, which was not statistically different from the control.

This suggests that the combined stress of nitrogen limitation and elevated salinity may have exceeded the optimal physiological tolerance of the alga, diverting metabolic resources away from canthaxanthin biosynthesis and toward essential survival mechanisms – a phenomenon also reflected in reduced biomass. TAP medium with nitrogen reduction alone showed the lowest total carotenoid production at 770.77 ± 219.41 µg.

The combined effects of salt and nitrogen stress have been shown to significantly enhance carotenoid accumulation in various microalgae. For example, *Tetraedron minimum* exhibited a nearly 5-fold increase in secondary carotenoids such as astaxanthin and adonixanthin when exposed to both salt addition and nitrogen starvation (Doppler et al. 2021). Nitrogen plays a critical role in cellular metabolism, and its limitation often results in nutrient deficiency and reduced algal growth. The present study supports this relationship: *Chlorosarcinopsis* PY02 grown under 50% nitrogen reduction in TAP medium – especially when combined with salt stress – showed a notable decrease in biomass compared with control conditions. Similar trends have been reported in *H. pluvialis* and *D. tertiolecta*, where nitrogen limitation also suppressed growth (Huang et al. 2019; Shin et al. 2015). Conversely, Cherdchukeattisak et al. (2018) reported significantly higher biomass in *Chlorosarcinopsis* PY02 under similar nitrogen-reduced conditions. This discrepancy may be attributed to differences in culture volume, underscoring the influence of cultivation scale on biomass and carotenoid production in *Chlorosarcinopsis* PY02.

Despite reduced growth, nitrogen limitation is a well-established trigger for carotenoid biosynthesis in several microalgae. Clear increases in ketocarotenoids under nitrogen stress have been documented for *H. pluvialis, Chromochloris zofingiensis*, and *Scenedesmus rubescens* (Li et al. 2022; Chen et al. 2017; Jo et al. 2020). Similarly, salt-induced stress has been shown to elevate ketocarotenoid levels – including canthaxanthin and astaxanthin – in *H. pluvialis* and *C. zofingiensis* (Li et al. 2022; Mao et al. 2020). Other stress-tolerant species, such as *Trentepohlia aurea* and *Cephaleuros*, also exhibit increased β-carotene accumulation under adverse conditions (Chen et al. 2015; Brocklehurst et al. 2024).

In this study, salt supplementation proved more effective than nitrogen reduction alone in enhancing carotenoid production – particularly canthaxanthin – in *Chlorosarcinopsis* PY02. Although the role of nitrogen deprivation in promoting carotenoid accumulation varies across species, several studies support its complementary effect (Lemoine and Schoefs 2010; Shi et al. 2020; Oslan et al. 2021).

Overall, stress-induced carotenoid accumulation is a well-recognized phenomenon in algae subjected to salinity or nutrient limitation. Although these conditions may suppress growth, they can stimulate the synthesis of secondary carotenoids, resulting in higher pigment yields. The present findings support the use of simple, low-cost chemical stressors such as NaCl as effective tools for promoting both biomass and carotenoid production in *Chlorosarcinopsis* PY02, offering a promising strategy for industrial-scale microalgal bioprocesses aimed at natural pigment production (Ren et al. 2021; Li et al. 2022; Bleisch et al. 2025).

The spot-test technique employed in this study provided a simple and rapid visual method for screening pigment induction under different stress conditions. However, it has limitations for scale-up applications or for precise growth quantification. We emphasize that future studies – particularly those aimed at process scale-up – should incorporate more rigorous inoculum standardization (e.g., optical density measurements, cell counts, or DW) to ensure high reproducibility across experiments.

In summary, this study highlights the critical influence of environmental factors, particularly salinity and nutrient availability, in optimizing algal cultivation for enhanced biomass and carotenoid production. These findings pave the way for future research aimed at elucidating the regulatory mechanisms involved and advancing cultivation strategies for commercial algal biotechnology.

## Conclusions

This study highlights the importance of optimizing chloride salt concentrations in the culture medium to enhance biomass production and carotenoid synthesis in *Chlorosarcinopsis* PY02. Among the tested conditions, salt supplementation alone proved to be the most effective strategy for increasing the production of valuable antioxidant compounds, particularly canthaxanthin. The combination of salt and nitrogen stress did not produce a synergistic effect on canthaxanthin levels, indicating that although the alga is stress-tolerant, a balanced degree of abiotic stress is essential for optimizing pigment yield. These findings contribute to a deeper understanding of algal stress responses and their implications for pigment biosynthesis. The demonstrated effectiveness of chloride-induced stress offers a practical, low-cost strategy for boosting carotenoid yields, with potential applications in pharmaceuticals, nutraceuticals, and other biotechnology sectors. By presenting a simple and scalable cultivation approach, this study provides a foundation for future efforts aimed at industrial-scale production of microalgae-derived bioactive compounds.
